# Specification of self-reported late-term impairments 3–7 years after primary breast cancer treatment: a nationwide cross-sectional study among Danish breast cancer survivors

**DOI:** 10.1007/s11764-025-01815-3

**Published:** 2025-05-06

**Authors:** Kim Michéle Feder, Kim Gordon Ingwersen, Hans Bjarke Rahr, Heidi Klakk Egebæk, Lonny Stokholm, Marianne Djernes Lautrup

**Affiliations:** 1 Department of Physiotherapy, Vejle Hospital, University Hospital of Southern Denmark, Beriderbakken 4, 7100 Vejle, Denmark; 2 Department of Regional Health Research, University of Southern Denmark, Campusvej 55, 5230 Odense, Denmark; 3 Department for Applied Research and Development, University College South Denmark (UC SYD), Degnevej 16, 6705 Esbjerg, Denmark; 4 OPEN, Open Patient data Explorative Network, J.B. Winsløws Vej 21, 3 Floor, 5000 Odense, Denmark; 5 Department of Surgery, Vejle Hospital, University Hospital of Southern Denmark, Beriderbakken 4, 7100 Vejle, Denmark; 6 Department of Clinical Research, University of Southern Denmark, Winsløwparken 19, 3 Floor, 5000 Odense, Denmark; 7 Department of Plastic and Breast Surgery, Aarhus University Hospital, Palle Juul-Jensens, Boulevard 35, 8200 Aarhus, Denmark

**Keywords:** Danish breast cancer survivors, Self-reported late-term impairments, Nationwide questionnaire, Registry extract, The Danish secondary healthcare

## Abstract

**Purpose:**

To describe the characteristics of Danish women treated for primary breast cancer, the prevalence and severity of self-reported late-term impairments, and the registration of these impairments in the Danish National Patient Registry.

**Methods:**

This is a nationwide cross-sectional survey study. A nationwide sample of 9927 women were invited to complete a questionnaire on late-term impairments, including shoulder impairment, lymphedema, fatigue, and chemotherapy-induced neuropathy. Severity was scored on validated patient-reported scales. Clinical characteristics of women and diagnostic codes for “late-term effects” were extracted from the Danish National Patient Registry.

**Results:**

The response rate was 60.9%. The 6046 responders were on average 57 years old at surgery, and 53.5% had a lower education level, 62.7% were married, 56.7% had a body mass index ≥ 25, and 54.4% had one or more co-morbidities. Overall, 60.7% reported having late-term impairments from their breast cancer treatment. The most common impairments were shoulder impairment (75.3%), fatigue (56.9%), chemotherapy-induced neuropathy (49.6%), and lymphedema (26.3%). On average, 58.0% women reporting impairments scored moderate to severe disturbances on validated patient-reported scales. Despite the high self-reported rates, impairments were rarely recorded in the Danish National Patient Registry (lymphedema, 1.3%; fatigue, 0.2%; shoulder impairment, 0.1%; and chemotherapy-induced neuropathy, 0.1%).

**Conclusion:**

More than 60% of Danish breast cancer survivors reported moderate to severe late-term impairments 3–7 years post-treatment, yet these impairments were rarely recorded in the Danish National Patient Registry.

**Implications for Cancer Survivors:**

Future research should focus on organizational structures in the Danish secondary healthcare, in relation to facilitating screening and timely detection of late-term impairments.

## Introduction

Breast cancer is the most prevalent cancer among women worldwide, with nearly 2.3 million new cases reported in 2022 [1]. In Denmark, approximately 5000 women are diagnosed with breast cancer each year [2]. Mammography screening, along with advanced improved surgical and oncological treatments [3], has increased the 5-year survival rate for breast cancer in Denmark to 90% [4]. The improved survival increases the importance of focusing on these women’s long-term quality of life and long-term impairments after treatment. Among the latter, upper limb late-term impairments (e.g., persistent or long-lasting health problems that develop as a delayed consequence of breast cancer treatment) remain common [5–8], particularly, shoulder impairment such as functional disabilities and decreased range of motion (ROM) (50%) [5, 6], lymphedema (27%) [5, 7], persistent pain like chemotherapy-induced neuropathy (30%) [5], and fatigue (30%) [5, 8]. These impairments have been shown to have a negative impact on quality of life (QoL) including physical, psychological, and social domains [9, 10]. However, previous studies often included a small and random sample size or focused separately on the risk of a specific surgical method, e.g., axillary lymph node dissection on a single late-term impairment, e.g., lymphedema [5]. In fact, no studies included a broad view on different late-term impairments at the same time [5–8].

Furthermore, despite a high prevalence of up to 50% late-term impairments up to 10 years after treatment [5, 6], little is known about the prevalence and severity of self-reported late-term impairments as judged by validated scales in Denmark. Lastly, since standard evaluation or treatment of late-term impairments in Denmark is not routinely offered to breast cancer survivors, it is unknown to what extent the Danish secondary healthcare system [11] (specifically, Danish hospitals) systematically takes care of late-term impairments.

This study aimed (1) to describe the clinical and socio-demographic characteristics of Danish women treated for primary breast cancer, (2) to describe the nationwide prevalence and severity of common self-reported late-term impairments, and (3) to elucidate the extent to which these impairments are registered in the Danish National Patient Registry.

## Methods

### Study design

This study was a nationwide cross-sectional survey study among Danish primary breast cancer survivors, including information from high-quality Danish national registries. Reporting followed the “Strengthening the Reporting of Observational Studies in Epidemiology” (STROBE) guideline [12].

### Setting

Based on data from the Danish Civil Person Registration (CPR) system and the Danish National Patient Registry (DNPR), the study population was identified by the Danish Health Data Agency. The CPR system includes all personal information, e.g., date of birth, marital status, death, and current residence (Denmark/Not Denmark) [13]. The DNPR includes the International Classification of Diseases, 10 th version (ICD10) [14, 15] of diagnostic codes, procedures, and treatments codes of all hospital contacts in Denmark [14]. Due to the nature of the Danish tax–funded healthcare system, it is mandatory to register all healthcare services in the DNPR when the woman has any contact with the healthcare system [14], including breast cancer treatments.

The women extracted by the Danish Health Data Agency were electronically invited to participate in a nationwide questionnaire on late-term impairments, regarding shoulder impairment, lymphedema, fatigue, and chemotherapy-induced neuropathy. The questionnaires were sent out from Vejle Hospital, University Hospital of Southern Denmark from March 3, 2022, to May 5, 2022, including a first reminder on March 7, 2022, and a second reminder on March 11, 2022. Access to the questionnaire was ended on May 5, 2022.

### Participants

Women were eligible to participate in the nationwide questionnaire, if they had undergone surgery for primary breast cancer (ICD10 C50*), breast-conserving surgery (BCS) (procedure codes: KHAB40, KHAB40 A), or mastectomy (procedure codes: KHAC20, KHAC25), including sentinel lymph node biopsy (SLNB) (procedure code: KPJD42 C) or axillary lymph node dissection (ALND) (procedure codes: KPJD42, KPJD52) between January 1, 2015, and December 31, 2019, and if they were between 18 and 71 years old at the time of surgery.

Due to the risk of double exposure leading to late-term impairments, women were excluded, if they had undergone bilateral breast cancer surgery (supplementary codes: TUL3 (double-sided) or coded with TUL1 (right sided) and TUL2 (left sided)), if they underwent primary or secondary breast reconstruction at any time (procedure codes: KGAE, KGAE50, KGAF, KHAC10, KHAC15, KHAD99 A, KHAE, KHAE00, KHAE05, KHAE10, KHAE20, KHAE25, KHAE99, KHAE99 A, KHAE99B, KHAE99 C), if the tumor was fixed to the thoracic/chest wall (supplementary codes: AZCD16, AZCD16a) or had spread outside of the breast and/or axilla (supplementary code: AZCD41), and if women had a cancer relapse (diagnostic code: DZ031RR; procedure code: KHAF00; supplementary codes: ZDW51E, ZDW51 F).

### Responders and non-responders

Women were categorized as responders when a complete or partly completed questionnaire returned. Non-responders included women who were invited but did not reply.

### Data sources and measurement

#### The questionnaire

The questionnaire was a self-administered questionnaire delivered via REDCap® [16] in three separate sections: *Sect. 1*: demographic and socio-demographic questions; *Sect. 2:* questions regarding breast cancer–related late-term impairments in four dimensions: shoulder impairment, lymphedema, fatigue, and chemotherapy-induced neuropathy; and *Sect. 3:* validated patient-reported scales (QuickDASH, LYMF-ICF-DK, and BFI) for assessing the severity.

The questionnaire was developed by the research group, which included experts within questionnaire development, breast surgeons, and oncological physiotherapists. Data quality was ensured through range limitations in questions with entering numeric values and checkbox solutions, when answers were entered in REDCap® [16].

The questionnaire was tested and completed by the research group to correct typing errors, technical functions of automatic data validation, and branching. Subsequently, a panel of eight persons from the Patient and Relatives Council (PPR) from Vejle Hospital, University Hospital of Southern Denmark evaluated the functions, timeframe, and content and specifically evaluated if anything could be offensive to someone who had breast cancer. Their feedback took place in writing and further in person or by phone conversation for further clarification. Based on their feedback, the self-administered questionnaire was refined for clarifications, and a more user-friendly layout was designed.

#### Self-reported characteristics of breast cancer survivors

Highest attained educational level (short, medium, long), employment (salaried or self-employed, sick leave, retired, other status), co-habitation status (living alone, living together), and children (no children/pregnant for the first time, children living at home, children living on their own, children living at home and living on their own), body mass index (BMI) calculated from height and weight (kg/m^2^), alcohol consumption (0 units/week, 1–7 units/week, 8–14 units/week, ≥ 15 units/week), smoking habits (nonsmoker, previous smoker, current smoker), and co-morbidities (see below) were self-reported information.

The following self-reported co-morbidities include allergy (not asthma), hypertension, cardiovascular disease, neurological disorder, chronic lung disease, diabetes (type 1 or 2), osteoporosis, migraine or frequent headaches, herniated disc, back/neck disorder, osteoarthritis, rheumatoid arthritis, multiple sclerosis, mental disorders and/or stress (less than 6 months), and mental disorders and/or stress (more than 6 months). The co-morbidity variable was classified by numbers of co-morbidities (besides breast cancer) as 0, 1, 2, and 3 +.

Additional self-reported variables were collected: whether or not the affected arm was the dominant arm used in daily life; whether pain medication was taken for late-term impairments, as well as type and frequency; and whether the women had resigned from work due to shoulder late-term impairments.

#### Distribution of questionnaire and data storage

Each person in Denmark has access to a secure digital mail account (e-Boks) [17]. However, exemptions can occasionally be granted to elderly citizens, allowing them to opt out of using e-Boks. An invitation to participate in the nationwide cross-sectional survey study was sent to their e-Boks, including a link to the questionnaire. Women without an e-Boks were not invited.

The questionnaire was hosted on a secure server by the Open Patient Data Explorative Network (OPEN). All answers collected in this study were treated and stored strictly confidentially in a Research Electronic Data Capture (REDCap®) database [16]. Each participant was labeled with an ID number in the REDCap® database to ensure pseudo-anonymity. After the nationwide cross-sectional study was completed, all data were subsequently stored and handled on the Danish Health Authorities secure server (FSEID-00005920), to comply with current data protection standards [18].

### Covariates

#### Register-based characteristics of breast cancer survivors

Age at the time of surgery (median with interquartile range (IQR)), geographical place of treatment (Capital Region of Denmark, Region Zealand, The North Denmark Region, Central Denmark Region, Region of Southern Denmark), type of surgical treatment (BCS, mastectomy, SLNB, ALND, +/− radiotherapy), complications (skin necrosis, wound infection, hematoma, seroma puncture), type of oncological treatment (chemotherapy, endocrine therapy), received physiotherapy during their breast cancer treatment (yes/no), and marital status (single, married, divorced, widow) were obtained from the register data.

### Self-reported late-term impairments

The prevalence of self-reported late-term impairments 3–7 years after primary breast cancer treatment was shoulder impairment, lymphedema, fatigue, and chemotherapy-induced neuropathy. Additionally, participants were asked to identify their two most bothersome late-term impairments among these conditions.

### Validated patient-reported scales

The severity of shoulder impairment, lymphedema, and fatigue was reported using QuickDASH, LYMF-ICF-DK, and BFI.

The *Quick-Disabilities of Arm, Shoulder and Hand (QuickDASH)* is a patient-reported region-specific questionnaire and a valid and reliable outcome measure for shoulder and other upper extremity assessments within the last week [19, 20]. The QuickDASH was used among all women who participated in this nationwide questionnaire. The 11 items (three for symptoms, eight for function) are scored on a Likert numeric rating scale ranging from 1 (no difficulty/symptoms) to 5 (extreme difficulty/unable to do symptoms) [19–21]. Each item score is equally weighted and added to a total percentage score. The higher the score, the greater the patient-reported impairment is [21]. In general, there is no specification on the score division. Therefore, in this study, the QuickDASH score was classified as follows, based on an average baseline score among individuals with persistent shoulder pain from trials with similar demographic distributions as this study population [22, 23]: < 20 (mild discomfort in the upper extremities), ≥ 20–40 (moderate discomfort in the upper extremities), and > 40 (severe discomfort in the upper extremities).

The *Lymphedema Functioning, Disability and Health (LYMF-ICF-DK)* questionnaire is valid and reliable for assessing self-reported impairments in Danish patients with breast cancer–related arm lymphedema [24, 25]. In this study, the first seven questions from the LYMF-ICF-DK, corresponding to domain 1, were used to assess the degree of lymphedema and its impact on impairments in physical function [25], among all women who participated in this nationwide questionnaire. The domain is scored on a numeric rating scale ranging from 0 (not at all) to 10 (a lot) and summed to a total percentage score [24, 25]. According to the World Health Organization (WHO) taxonomy, impairments within the domain can be classified as: < 25 (mild impairments in physical function), ≥ 25–50 (moderate impairments in physical function), and > 50 (severe impairments in physical function) [24, 25].

The *Brief Fatigue Inventory (BFI)* questionnaire is valid and reliable to assess the severity of cancer-related fatigue and the impact on daily functioning in the past 24 h among patients with fatigue due to cancer treatment [26]. The BFI was used among all women who participated in this nationwide questionnaire. The BFI consists of nine questions answered using an 11-point rating scale (0 = no fatigue, 10 = the most severe level of fatigue) [26–28]. Each item score is equally weighted and added to a total percentage score. The higher the score, the greater the patient-reported fatigue, and the score is divided as the following BFI score: < 40 (mild fatigue), ≥ 40–80 (moderate fatigue), and > 80 (severe fatigue) [27, 28].

### Registration in the DNPR

The registration of diagnostic codes for “late-term effects” in the Danish secondary healthcare was taken from the DNPR for shoulder impairment (T983D4 = chronic pain after cancer treatment or T983DB = symptoms of musculoskeletal conditions after cancer treatment), for lymphedema (I972 = lymphedema after mastectomy or T983DA = lymphedema after cancer treatment), for fatigue (T983D5 = fatigue after cancer treatment), and for chemotherapy-induced neuropathy (T983DD = neuropathy after cancer treatment).

### Statistical methods

#### Descriptive statistical methods

This study is a descriptive study, where data are presented descriptively as counts and percentages for categorical variables and medians and interquartile range (IQR) for continuous variables.

When height and weight were beyond 4.5 SDs (standardized for age and height/weight) [29], BMI scores were replaced with missing values. Other missing values were reported in the tables by indicating how many missings there were for each of the questionnaires.

All management and descriptive statistics were performed using STATA 18.0 (Statacorp LP, College Station, TX, USA) software.

## Results

### Participants: responders and non-responders

**Fig. 1:**
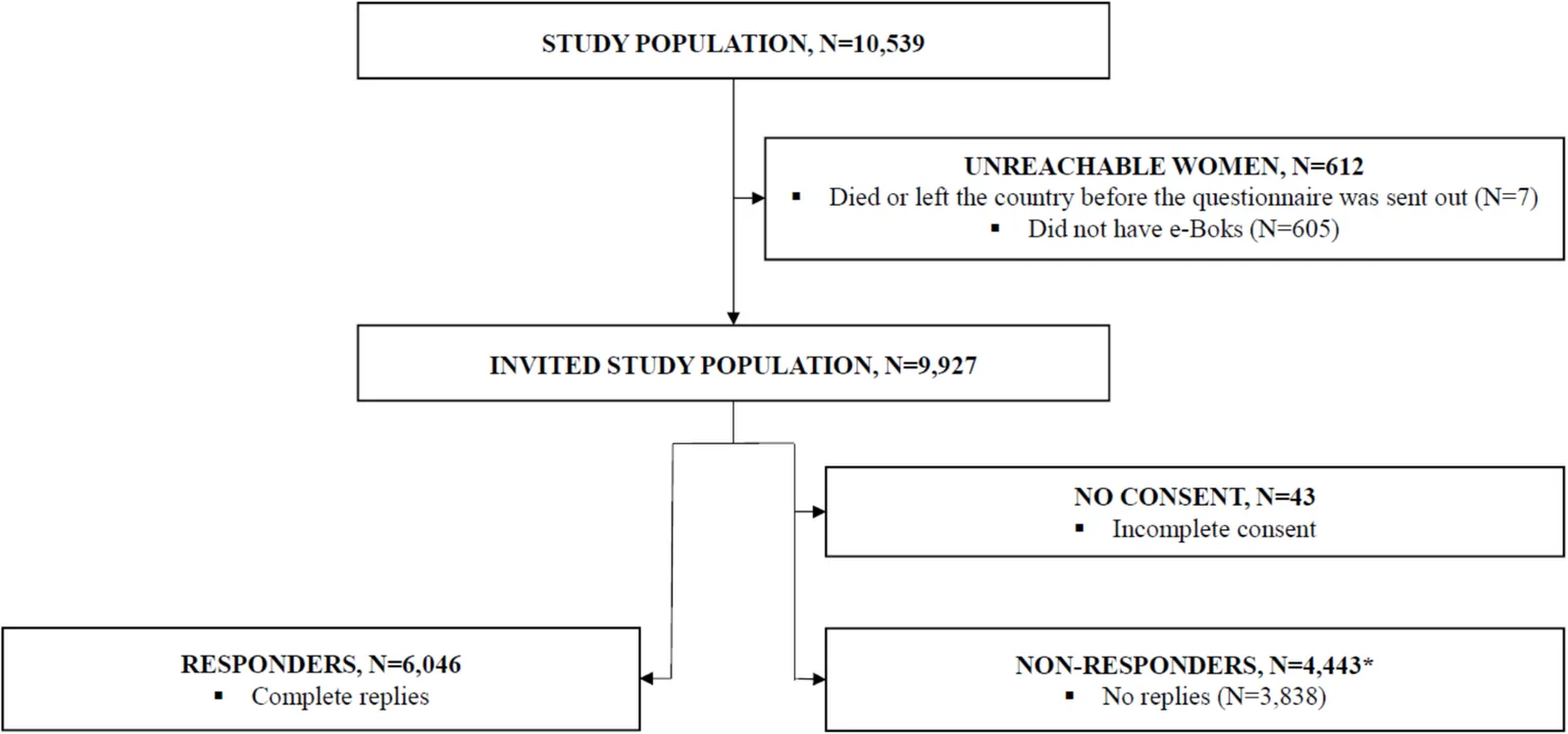
Flowchart over responders and non-responders among Danish breast cancer survivors. *A total of 4443 women are included as non-responders, because it is not possible on the Danish Health Authorities secure server (FSEID-00005920) to separate women who did not reply from those who did not have e-Boks

The Danish Health Data Agency identified a nationwide study population consisting of 10,539 eligible women, while 612 women were unreachable due to death, having left the country or not having an e-Boks. Therefore, 9927 women were invited to participate in the nationwide cross-sectional survey study. Of these, 3838 women did not reply to the invitation, while 43 women were excluded due to incomplete consent. In total, 6046 women responded, yielding a response rate of 60.9%, and were included in the descriptive analysis (Fig. 1).

#### Responders vs. non-responders

Responders and non-responders were not different from each other, except for their marital status. Non-responders had a higher percentage of divorced or widows (Table 1).

**Table 1:** The characteristics of women treated for primary breast cancer based on clinical information, socio-demographics, lifestyle, and health information

Characteristics	Responders	Non-responders
	*N* = 6046	*N* = 4443
**Clinical information**		
*Age at time of surgery—median (IQR)*^a^*, n (%)*	57.0 (45.1–68.9)	56.5 (43.7–69.2)
≥ 18–39 years	256 (4.2)	218 (4.9)
40–49 years	917 (15.2)	772 (17.4)
50–59 years	2043 (33.8)	1413 (31.8)
60–71 years	2830 (46.8)	2040 (45.9)
*Geographical place of treatment—region, n (%)*^a^		
Capital Region of Denmark	1759 (29.1)	1556 (35.0)
Region Zealand	999 (16.5)	720 (16.2)
The North Denmark Region	645 (10.7)	539 (12.1)
Central Denmark Region	1410 (23.3)	946 (21.3)
Region of Southern Denmark	1233 (20.4)	682 (15.3)
*Type of surgery and radiotherapy (standard clinical guidelines), n (%)*^a^		
BCS & SLNB + radiotherapy^b^	3141 (52.0)	2174 (48.9)
BCS & ALND + radiotherapy^b^	699 (11.6)	493 (11.1)
Mastectomy & SLNB + radiotherapy^b^	691 (11.4)	533 (12.0)
Mastectomy & SLNB − radiotherapy^b^	683 (11.3)	529 (11.9)
Mastectomy & ALND + radiotherapy^b^	239 (4.0)	214 (4.8)
BCS & SLNB − radiotherapy or BCS & ALND − radiotherapy^c^	432 (7.1)	371 (8.4)
SLNB or ALND + radiotherapy, or only radiotherapy^d^	161 (2.7)	129 (2.9)
*Occurred complications, n (%)*^a^		
Skin necrosis, n	< 5 (n/a)	< 5 (n/a)^g^
Wound infection, n	14 (0.2)	16 (0.4)
Hematoma, n	18 (0.3)	15 (0.3)
Seroma (≥ 5 seroma punctures), n	36 (0.6)	33 (0.7)
*Received chemotherapy, n (%)*^a^	3,596 (59.5)	2,511 (56.5)
*Received endocrine therapy, n (%)*^a^	5,272 (87.2)	3,819 (86.0)
*Received physiotherapy, n (%)*^a^	3,415 (56.5)	2,413 (54.3)
**Socio-demographics**		
*Marital status, n (%)*^a^		
Single	786 (13.0)	599 (13.5)
Married	3791 (62.7)	2281 (51.3)
Divorced	1003 (16.6)	928 (20.9)
Widow	466 (7.7)	428 (9.6)
Missing	0 (0.0)	207 (4.7)
*Highest attained education level, n (%)*^e^		
Short	3232 (53.5)	
Medium	2019 (33.4)	
Long	734 (12.1)	
Missing	61 (1.0)	
*Employment, n (%)*^e^		
Employed for wages or self-employed	2577 (42.6)	
Sick leave	168 (2.8)	
Retired	2902 (48.0)	
Other status	399 (6.6)	
*Co-habitation status, n (%)*^e^		
Living alone	1674 (27.7)	
Living together	4155 (68.7)	
Missing	217 (3.6)	
*Children, n (%)*^e^		
No children/pregnant for the first time	635 (10.5)	
Children living at home	757 (12.5)	
Children living on their own	4123 (68.2)	
Children living at home and living on their own	420 (6.9)	
Missing	111 (1.9)	
**Lifestyle & health**		
*Body Mass Index (BMI) (kg/m*^2^*), n (%)*^e^		
≤ 18.5 (underweight)^f^	79 (1.3)	
18.5–24.9 (normal weight)^f^	2457 (40.6)	
25.0–29.9 (pre-obesity)^f^	2,004 (33.1)	
30.0–34.9 (obesity—class I)^f^	973 (16.1)	
35.0–39.9 (obesity—class II)^f^	314 (5.2)	
≥ 40 (obesity—class III)^f^	141 (2.3)	
Missing	78 (1.3)	
*Alcohol consumption, n (%)*^e^		
Drinking no alcohol	2280 (37.7)	
1–7 units per week	2967 (49.1)	
8–14 units per week	676 (11.2)	
≥ 15 units per week	83 (1.4)	
Missing	40 (0.6)	
*Smoking habits, n (%)*^e^		
Non smoker	3,135 (51.9)	
Previous smoker	659 (10.9)	
Current smoker	2231 (36.9)	
Missing	21 (0.3)	
*Co-morbidities, n (%)*^e^		
No co-morbidities	2759 (45.6)	
1	1171 (19.4)	
2	973 (16.1)	
3 +	1143 (18.9)	

^a^Register-based information

^b^BCS = breast-conserving surgery, SLNB = sentinel lymph node biopsy, ALND = axillary lymph node dissection, + radiotherapy = received radiotherapy, − radiotherapy = did not received radiotherapy

^c^Women who received this combination are not meeting the standard clinical guidelines for breast cancer treatment [3] but are a part of a randomized controlled trial led by the Danish Breast Cancer Cooperative Group (DBCG). DBCG is a Danish organization that determines the clinical guidelines within breast cancer, and with this trial, it aims to possibly supplement the existing standard clinical guidelines [30]

^d^Women who received one of this combinations are not meeting the standard clinical guidelines for breast cancer treatment and were categorized as misregistered in the DNPR

^e^Self-reported information

^**f**^WHO’s definition of BMI categorizing [31]

^**g**^Not applicable

### Descriptive data

#### Characteristics of the responders

The median time from surgery to questionnaire completion was 4.6 years. The average age among the responders was 57 years at time of surgery. The women were more frequently treated with surgery in the Capital Region of Denmark (29.1%) or Central Denmark Region (23.3%). The majority underwent a BCS and SLNB (52.0%) on the left (51.1%) or right (47.4%) side. The proportion of women who started with a BCS but ended up with a mastectomy was 15.1%. Furthermore, women reported ≤ 1.1% complications after surgery, 59.5% received chemotherapy, 87.2% received endocrine therapy, and 56.5% received physiotherapy during their breast cancer treatment (Table 1).

The majority of respondents had a lower education level (53.5%), were married (62.7%), and were retired from work (48.0%), with children living on their own (68.2%). Furthermore, the majority of the responders reported having a BMI ≥ 25 (56.7%), drinking 1–7 units of alcohol per week (74.5%), being non-smokers (51.9%), and having one or more co-morbidities (54.4%) (Table 1).

#### Self-reported late-term impairments (prevalence and severity) and DNPR diagnostic registrations

Among the responders, 60.7% reported late-term impairments (Table 2), of which 54.6% indicated that their impairments on breast cancer treatment are not on the dominant arm they use in daily life.

**Table 2:** An overview of the nationwide prevalence and severity of common self-reported late-term impairments (overall and severe), the registration of these impairments recorded in the Danish Registry, and the combinations of the two bothersome self-reported late-term impairments

Responders						*N* = 6046
**Self-reported late-term impairments, ***n* **(%)**						
Yes						3667 (60.7)
No						2244 (37.1)
*Missing*						*135 (2.2)*
**Type of self-reported late-term impairments**						*N* = 3667
			**Overall**^a^	**Severe**^b^		**DNPR diagnosis**^c^
			*N* = 3667	*N* = 3667		*N* = 6046
**Shoulder impairment,** *n*** (%)**			2762 (75.3)	1946 (53.1)		9 (0.1)^c^
QuickDASH score, *n* (%)						
< 20 (mild discomfort in the upper extremities)			1363 (49.3)	967 (49.7)		
≥ 20–40 (moderate discomfort in the upper extremities)			812 (29.4)	536 (27.5)		
> 40 (severe discomfort in the upper extremities)			587 (21.3)	443 (22.8)		
**Fatigue**, *n* (%)			2085 (56.9)	1520 (41.5)		10 (0.2)^c^
BFI score, *n* (%)						
< 40 (mild fatigue)			846 (40.6)	612 (40.3)		
≥ 40–80 (moderate fatigue)			1049 (50.3)	781 (51.4)		
> 80 (severe fatigue)			190 (9.1)	127 (8.4)		
**Chemotherapy-induced **neuropathy, *n* (%)			1818 (49.6)	902 (24.6)		7 (0.1)^c^
No score						
**Lymphedema**, *n* (%)			965 (26.3)	538 (14.7)		78 (1.3)^c^
LYMPH-ICF-DK score—domain 1, n (%)						
< 25 (mild impairments in physical function)			356 (36.9)	194 (36.1)		
≥ 25–50 (moderate impairments in physical function)			308 (31.9)	160 (29.7)		
> 50 (severe impairments in physical function)			301 (31.2)	184 (34.2)		
**Combinations of the two bothersome self-reported late-term impairments**^d^, *n* (%)						*N* = 3667
	Shoulder impairment	Lymphedema	Chemotherapy-induced neuropathy		Fatigue	Missing
Shoulder impairment	974 (26.6)	193 (5.3)	318 (8.7)		461 (12.6)	
Lymphedema		160 (4.4)	66 (1.8)		119 (3.2)	
Chemotherapy-induced neuropathy			281 (7.7)		237 (6.5)	
Fatigue					703 (19.2)	
Missing						155 (4.2)

^a^Indicates the woman’s overall self-reported late-term impairments, where the women can be included several times under the four listed types of late-term impairments

^b^Indicates woman’s primary two most bothersome self-reported late-term impairments, where the women can be included at most twice

^c^Indicates the registration of diagnostic codes for “late-term effects” in the Danish secondary healthcare, taken from the DNPR. For shoulder impairment = T983D4 (chronic pain after cancer treatment) and T983DB (symptoms of musculoskeletal conditions after cancer treatment), for lymphedema = I972 (lymphedema after mastectomy) and T983DA (lymphedema after cancer treatment), for fatigue = T983D5 (fatigue after cancer treatment), and for chemotherapy-induced neuropathy = T983DD (neuropathy after cancer treatment)

^d^Distributed among the individual women

Out of the 3667 women, the prevalence of common self-reported late-term impairments was shoulder impairment (75.3%), fatigue (56.9%), chemotherapy-induced neuropathy (49.6%), and lymphedema (26.3%). As the women were asked to indicate the two most bothersome late-term impairments among these four, respondents still ranked shoulder impairment (53.1%) and fatigue (41.5%) as one of their two most bothersome late-term impairments followed by chemotherapy-induced neuropathy (24.6%) and lymphedema (14.7%). The most common constellation of bothersome late-term impairments was shoulder impairment and fatigue (12.6%) or shoulder impairment and chemotherapy-induced neuropathy (8.7%) (Table 2).

Among women reporting one or two bothersome impairments, 50.3% with shoulder impairment had a QuickDASH score of ≥ 20, indicating moderate to severe upper extremity impairment. Similarly, 59.8% of those with fatigue had a BFI score of ≥ 40, reflecting moderate to severe fatigue, and 63.9% with lymphedema scored ≥ 25 on the LYMPH-ICF-DK, indicating moderate to severe physical function impairments (Table 2).

Registration in the DNPR of specific “late-term effects” related to breast cancer treatment was only present for a small minority of breast cancer survivors: lymphedema, 1.3%; fatigue, 0.2%; shoulder impairment, 0.1%; and chemotherapy-induced neuropathy, 0.1% (Table 2).

On average, 35.3% of all responders who experienced moderate to severe late-term impairments indicated pain medication consumption. The majority took prescription medicine 4–7 days per week (Table 3). In addition, for shoulder impairment, 13.6% of 50–59 year olds responded that they had to resign from work due to shoulder late-term impairments in the age of 50–59 years.

**Table 3:** Indicated consequences on bothersome self-reported late-term impairments

	Shoulder impairment	Fatigue	Chemotherapy-induced neuropathy	Lymphedema
	*N* = 1946	*N* = 1520	*N* = 902	*N* = 538
**Pain medication due late terms—yes,** *n* (%)	667 (34.3)	595 (39.1)	336 (37.3)	160 (29.7)
Prescription medicine	250 (37.5)	227 (38.2)	142 (42.3)	66 (41.2)
Over the counter medicine	211 (31.6)	159 (26.7)	83 (24.7)	50 (31.2)
Both	206 (30.9)	209 (35.1)	111 (33.0)	44 (27.5)
4–7 days per week	384 (57.6)	350 (58.8)	204 (60.7)	99 (61.9)
1–3 days per week	220 (33.0)	189 (31.8)	103 (30.7)	45 (28.1)
Rarely	63 (9.4)	56 (9.4)	29 (8.6)	16 (10.0)

## Discussion

### Summary and interpretation

More than 60% of Danish breast cancer survivors reported late-term impairments within 3–7 years post-treatment. The majority of women reporting impairments scored moderate to severe disturbances on validated patient-reported scales and had a consumption of prescription pain medication 4–7 days/week. In addition, responders reporting shoulder impairment and aged between 50 and 59 years old had to resign from work due to their shoulder problems. Despite the high prevalence of impairments and the serious consequences for the women, specific diagnostic codes for “late-term effects” in the DNPR were only found in a very small proportion of breast cancer survivors. This suggests either an underutilisation of specialized treatment by these women, a negligence of registration of the relevant diagnostic codes in the Danish secondary healthcare, or that healthcare providers are not asking the right questions to screen for late-term impairments.

Our findings are also reflected in previous research, which demonstrates that the women often face significant long-term impairments, including physical and psychological impairments, greater use of medication (e.g., opioid analgesics), and challenges in returning to work [5–10, 32–34]. In addition, research highlights a lack of knowledge and emphasizes the need for greater efforts by healthcare providers and policymakers to recognize the need for screening and improve access to rehabilitation interventions to address these challenges [5, 9, 10, 32, 34].

Several reasons may contribute to the indicated insufficient attention given to late-term impairments of primary breast cancer treatment in Danish hospitals. Firstly, the primary objective of treatment in the Danish hospitals traditionally has been the cure of the patient, including follow-up programs for detection of cancer relapse [3, 35]. Secondly, there is a lack of evidence-based treatments to effectively address upper limb shoulder pain and impaired shoulder function related to the complexity of this late-term condition faced by women after breast cancer [5–10, 32, 34–37]. Lastly, many of these late-term impairments might fall beyond the typical scope of the oncologist’s expertise or focus. Addressing late-term impairments effectively necessitates enhanced collaboration and improved coordination in rehabilitation treatments, which may initially increase costs within the Danish secondary healthcare system. However, failing to prioritize these impairments could lead to significant societal costs in the future, including higher morbidity rates, increased medication use, and a reduced workforce [32–34].

Therefore, it is important to reconsider approaches on how to catch women in need for assistance regarding late-term impairments. Firstly, it is necessary to identify the need for screening for late-term impairments to help the treating physician identify the woman’s problem and hereby guide the most appropriate treatment [38]. Organizations for clinical practice, such as the Danish Health Authority, have recognized the need for screening tools for late-term impairments in their new Cancer Plan V (point 2) [39]. Secondly, the focus should be on the implementation of screening tools or instruments to enhance access to rehabilitation for cancer survivors [38]. The Danish Breast Cancer Group and Clinic Center for Late Effects (DCCL) in Denmark has just started developing a digital platform [40]. Thirdly, research initiatives should focus on developing and testing appropriate rehabilitation interventions to alleviate the late-term impairments. Fourthly, to secure sufficient surveillance of the prevalence of late-term impairments, increased focus on the correct use of diagnostic codes should be conveyed to clinical practice.

### Strengths and limitations

This study is one of the few evaluating self-reported late-term impairments among Danish breast cancer survivors 3–7 years post-treatment. A major strength is the size of this nationwide study with 6046 women participating and a response rate over 60% with little missing information (< 5%). Another strength is that the self-administered questionnaire explicitly is developed for Danish primary breast cancer survivors, including validated patient-reported scales such as QuickDASH, LYMF-ICF-DK, and BFI [19–21, 24–26], and is thoroughly tested by the research group and the panel from the Vejle Hospital Patient and Public advisory board before sending out. Furthermore, we consider it as strength that we through the unique Danish CPR system [13] could link questionnaire data with high-quality registry data [14], to ensure a high degree of completeness and accuracy.

A limitation of the current study includes the self-report of late-term impairments, i.e., no objective tests were included to verify the presence or severity of impairments. Furthermore, questionnaire studies in general have a risk of selection bias [41]. In the current study, the “healthy participant effect” could have an impact on our results, as it tends to be the most resourceful women, who participate in these types of study. This is also the case for 605 women who did not have e-Boks, which typically include the elderly and the sickest people. This bias may have caused an underestimation of the problem of late-term impairments among breast cancer survivors. However, this only strengthens our rationale of the need for a more structured system to identify women experiencing late-term impairments after breast cancer treatment.

### Generalizability

The responders in this study differed only slightly from the invited total study population (~ high external validity), where non-responders had a higher percentage of divorced or widowed individuals (Table 1). Our study reflects the Danish welfare system with a Danish tax–funded secondary healthcare [11, 14]. Therefore, our results may not be generalizable one-to-one to countries with different welfare systems than Denmark.

## Conclusion

Danish breast cancer survivors more frequently reported moderate to severe late-term impairments 3–7 years post-treatment, including consequences for medication consumption and their ability to work. In spite of that, these health problems were only recorded in the DNPR for a very small percentage of breast cancer survivors, suggesting that they may be overlooked by the Danish secondary healthcare. Future research should focus on organizational structures in the Danish secondary healthcare, in relation to facilitating screening and timely detection of late-term impairments.

## Data Availability

Data are not publicly available due to Danish law but are available from the corresponding author on reasonable request (https://orcid.org/0000-0003-3088-1833).
